# The Epidemiology of Primary Lateral Sclerosis: Results from a Population‐Based Cohort

**DOI:** 10.1002/ana.78105

**Published:** 2025-11-28

**Authors:** Rosario Vasta, Enrico Matteoni, Giorgio Pellegrino, Antonio Canosa, Umberto Manera, Francesca Palumbo, Maurizio Grassano, Sara Cabras, Alessandra Maccabeo, Fabrizio D'Ovidio, Gabriele Mora, Salvatore Gallone, Elisa D'Angelo, Letizia Mazzini, Fabiola De Marchi, Cristina Moglia, Adriano Chiò, Andrea Calvo

**Affiliations:** ^1^ ALS Center, Department of Neuroscience “Rita Levi Montalcini” University of Turin Turin Italy; ^2^ Neurology 1, AOU Città della Salute e della Scienza di Torino Turin Italy; ^3^ Institute of Cognitive Science and Technologies, National Research Council Rome Italy; ^4^ School of Advanced Studies, Center for Neuroscience University of Camerino Camerino Italy; ^5^ ALS Center, Department of Neurology Azienda Ospedaliero Universitaria Maggiore della Carità, and University of Piemonte Orientale Novara Italy

## Abstract

**Objective:**

In this population‐based study, we described the epidemiology of primary lateral sclerosis (PLS) in northern Italy and compared the clinical characteristics of patients with PLS to those with predominant upper motor neuron (PUMN) involvement and classic amyotrophic lateral sclerosis (ALS).

**Methods:**

Patients from the PARALS registry diagnosed with probable or definite PLS between 2007 and 2021 were included. Crude annual incidence rates were calculated, along with age‐ and sex‐specific rates. A survival analysis was performed to identify prognostic factors at diagnosis. Covariates included sex, age at onset, site of onset, diagnostic delay, forced vital capacity (FVC), change in ALS Functional Rating Scale (ΔFRS), and change in body mass index (ΔBMI).

**Results:**

A total of 57 PLS patients (2.7%) were included, with a crude incidence rate of 0.084 per 100,000 person‐years. Compared to PUMN and classic ALS, PLS patients were younger (median onset age 63.5 years, interquartile range [IQR] 54.9–70.4) and predominantly female (male‐to‐female ratio 0.58). Bulbar onset occurred in 11 cases (19.3%), all of whom later developed spinal symptoms. At censoring, 38 patients (66.7%) were still alive (median survival 8.3 years, IQR 5.7–12.3), corresponding to a point prevalence of 0.89 per 100,000. Survival was significantly associated with age at onset (hazard ratio [HR] 1.17, 95% confidence interval [CI]: 1.05–1.33, *p* = 0.001), male sex (HR 4.41, 95% CI: 1.24–15.6, *p* = 0.02), and FVC at diagnosis (HR 0.95, 95% CI: 0.93–0.98, *p* = 0.006).

**Interpretation:**

PLS was confirmed to be rarer than other ALS phenotypes. Patients had a higher age at onset than previously reported and a female predominance. Sex, age at onset, and respiratory function were key prognostic factors. ANN NEUROL 2026;99:606–613

Primary lateral sclerosis (PLS) is a degenerative disease characterized by symptoms of progressive upper motor neuron (UMN) dysfunction for at least 2 years.^1^ It is usually regarded as the UMN‐dominant end of the amyotrophic lateral sclerosis (ALS) spectrum, which encompasses progressive muscular atrophy at the opposite extreme and classic ALS at its center.^2^ However, PLS is accompanied by distinct demographic and clinical features that raise the question of whether it constitutes a separate disease entity.^1^, ^2^, ^3^, ^4^

Compared to ALS, people with PLS typically lack a familial history of the disease, with only rare exceptions reported,^5^, ^6^ and are usually younger at the time of onset.^1^, ^2^, ^3^, ^4^ Symptoms typically begin in the lower limbs, presenting as gait disturbances that impair balance and increase the risk of falls. Less commonly the disease has its onset in the bulbar region.^4^ Importantly, while electromyography may reveal only mild distal denervation changes in extremity muscles, other clinical or neurophysiological signs of lower motor neuron (LMN) involvement are absent.^1^

The progression of symptoms in PLS is generally slow. Eventually, respiratory muscle involvement may occur in advanced stages, occasionally necessitating mechanical ventilation. As a consequence, survival of patients with PLS is significantly longer than those with ALS, ranging from 7 to 14 years, with death often due to aspiration pneumonia, immobility‐related issues, or other comorbidities.^3^

Although this is the picture that has emerged from literature so far, no population‐based studies have been conducted to date. This limitation increases the risk of mischaracterization of the disease because of potential selection bias. Also, while some studies reporting an incidence of less than 0.1 per 100,000 person‐years,^4^ accounting for 2–5% of all incident motor neuron diseases,^2^, ^3^ the exact frequency of PLS remains uncertain.

Using a large population‐based cohort, here we describe the epidemiology and clinical characteristics of patients with PLS. To better understand the differences in clinical characteristics between patients with PLS and other motor neuron phenotypes, we compared patients with PLS with those with predominant UMN (PUMN) and classic ALS.

## Methods

The Piemonte and Valle d'Aosta ALS Register (PARALS) encompasses all patients who received a diagnosis within the ALS spectrum from 1995 onwards and who resided in Piemonte or Valle d'Aosta at the time of diagnosis.^7^ The vast majority of patients are seen at 1 of 2 ALS tertiary centers located in the cities of Torino and Novara. At both centers, patients are followed at regular intervals after diagnosis, with systematic collection of demographical, clinical and disease progression data. For the remaining patients, information is obtained from neurology departments across both regions.^7^

For the purpose of this study, we included patients from the PARALS register who were diagnosed between 2007 and 2021 with probable of definite primary lateral sclerosis (PLS), according to the most recent diagnostic criteria.^1^ We opted to also include patients who, at their first visit, had not yet reached 2 years of symptoms but subsequently met the definition of PLS during the study period (so called “early PLS” patients). Accordingly, for these patients the date of diagnosis was defined as the first visit during which PLS was suspected after excluding other etiologies. As such, all measures related to diagnosis (including diagnostic delay, survival, and disease progression) should be interpreted accordingly. All patients underwent a comprehensive diagnostic workup to exclude alternative conditions. This included brain and cervico‐thoracic magnetic resonance imaging, serological testing for Lyme disease and human T‐cell leukemia virus types 1 and 2, as well as assessments of serum copper and vitamin B12 levels. The majority of patients also underwent genetic testing for HSP‐related genes and for the 4 main ALS‐related genes (*C9orf72*, *SOD1*, *TARDBP* and *FUS*). None of the patients exhibited extrapyramidal signs.^4^

The crude incidence rate for each year of the study period was calculated by dividing the number of patients diagnosed in that specific year by the total population residing in Piemonte and Valle d'Aosta as of January 1st of the same year. Age‐ and sex‐specific incidence rates were also calculated. Additionally, to enable meaningful comparisons, an age‐ and sex‐adjusted incidence rate was calculated using the 2013 European standard population as the reference.^8^

To compare the demographic and clinical characteristics of patients with PLS to those with other ALS phenotypes, we also included patients diagnosed during the same period with either the PUMN or classic ALS forms, as these were previously defined.^9^

Several demographic and clinical variables were assessed and compared across these groups. Specifically, diagnostic delay was defined as the number of months from symptom onset to diagnosis, and the site of onset was categorized as bulbar, upper limbs, or lower limbs. Disease progression rate was calculated using the revised ALS Functional Rating Scale (ALSFRS‐r), both at the time of diagnosis [(48 – ALSFRS‐r score at diagnosis) divided by the diagnostic delay, ΔFRS at diagnosis] and at the end of follow‐up [(48 – ALSFRS‐r score at last visit) divided by the number of months from symptom onset to the last visit, ΔFRS at last visit]. The presence of respiratory symptoms at diagnosis was evaluated considering the ALSFRS‐r respiratory subscore; forced vital capacity (FVC) values at diagnosis were also compared. Change in body mass index (ΔBMI) at diagnosis was calculated as the difference between premorbid body mass index (BMI) and BMI at diagnosis, divided by the diagnostic delay. Biomarkers of muscle damage and mass, including serum levels of creatinine, creatine phosphokinase (CPK), phosphorus, and albumin^10^, ^11^ measured at the time of diagnosis, were also evaluated.

Comparisons of medians (reported with interquartile ranges [IQRs]) and proportions were performed using the Mann–Whitney *U* test and *χ* ^2^ test, respectively. In order to test whether the male‐to‐female ratio changed over time (see the Results section), a linear regression was computed within each phenotype considering the ratio as dependent variable and the year of diagnosis as independent variable.

Finally, a survival analysis was conducted. Survival was defined as the time from diagnosis to either death, tracheostomy, or the censoring date (set as December 31, 2024). A Cox proportional hazards regression model was developed to identify potential prognostic factors for PLS at the time of diagnosis. The model included sex, onset age, site of onset, diagnostic delay, FVC, ΔFRS, and ΔBMI at diagnosis as covariates.

The study was approved by the Ethical Committee of the Turin ALS Center (Comitato Etico Azienda Ospedaliero‐Universitaria Città della Salute e della Scienza, Torino, #0038876). Patients provided written informed consent.

## Results

A total of 2,110 patients with any ALS phenotype were included in the PARALS during the study period. Of these, 57 (2.7%) were diagnosed with PLS. This corresponded to an average annual crude incidence rate of 0.084/100,000 person‐years, that remained virtually unchanged (0.081/100,000 person‐years) when age‐ and sex‐adjusted for the European standard population. As expected for a very rare disease, the annual incidence rate showed considerable variability over the study period, although it remained stable when averaged across 5‐year intervals (Table S1 and Fig S1).

A subset of patients (17, 29.8.2%) underwent genetic testing for HSP‐related genes, although the testing protocols varied over time. Specifically, 13 patients were analyzed for both *SPG4* and *SPG7*, 5 for *SPG11*, 2 for *SPG15*, while 5 patients underwent more extensive genetic screening (Supplementary Materials). One patient carried a variant of uncertain significance (VUS) in the *BSCL2* gene.

The marital status and educational levels of PLS patients were comparable to those of individuals with PUMN and classic ALS. Similarly, smoking habits and the major comorbidities considered were evenly distributed across the 3 groups (Table S3).

**FIGURE 1:**
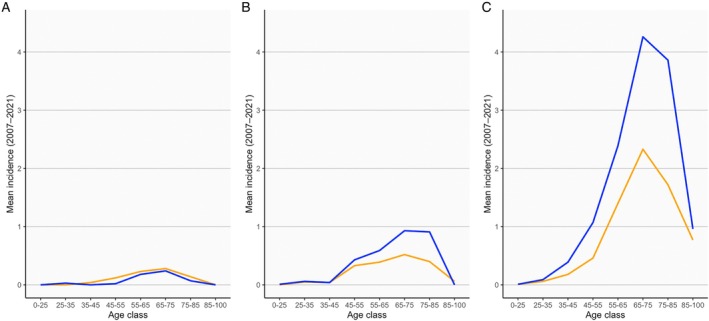
Age‐ and sex‐specific crude incidence rates across motor neuron phenotypes (A = primary lateral sclerosis; B = predominant UMN; C = classic ALS; blue = males; orange = females). ALS = amyotrophic lateral sclerosis; UMN = progressive upper motor neuron. [Color figure can be viewed at www.annalsofneurology.org]

The age at symptom onset was lowest in PLS patients (median 63.5 years), followed by those with PUMN (64.3 years) and classic ALS (66.9 years) (Table S3 and Fig 1). Notably, PLS patients were predominantly female, with a male‐to‐female ratio of 0.58. Correspondingly, the mean annual crude incidence rate was 0.104 per 100,000 person‐years in females and 0.064 in males. However, as lower motor neuron involvement increased, the sex distribution shifted, with the male‐to‐female ratio rising to 1.46 in PUMN and 1.64 in classic ALS (Table S3 and Fig 1). As expected, the diagnostic delay was longer (37.0 months, IQR 28.9–56.3) when early PLS patients were excluded. Spinal onset was the most common clinical presentation in PLS, although bulbar onset was observed in 11 cases (19.3%), with no significant demographic or clinical differences between the 2 subgroups (data not shown). However, all bulbar‐onset cases also exhibited spinal symptoms, either present at the first visit (7, 63.6%) or emerging during follow‐up (4, 36.4%). By definition, bulbar onset was not reported in PUMN or classic ALS.^9^

Among spinal‐onset PLS cases, symptoms typically began in the lower limbs (73.7%), often bilaterally (Table S3). The vast majority of these patients (40 cases, 86.9%) eventually developed bulbar symptoms over the course of the disease. Notably, only 1 patient who did not develop bulbar symptoms had a long‐term follow‐up, while follow‐up data were lacking for the remaining 5 patients (10.8%).

PLS was associated with slower disease progression, as indicated by a longer diagnostic delay which was nearly twice as long as in classic ALS (18.7 vs. 9.6 months). Reflecting this longer diagnostic delay, although the total ALSFRS‐r score at diagnosis in the PLS group was only slightly higher than in the other groups, their progression rate at that time was significantly lower, decreasing from 0.71 points/month in classic ALS to 0.61 in PUMN and 0.29 in PLS. This pattern was also reflected in muscle damage biomarkers: PLS patients showed significantly lower levels of serum CPK and phosphorus at diagnosis. In contrast, there were no significant differences in creatinine and albumin levels (Table S3). Notably, CPK and albumin were rarely abnormal in PLS patients (CPK was elevated in only 3 PLS patients, 8.6%, compared to 29, 22.0%, PUMN patients and 175, 39.9%, classic ALS patients; *p* < 0.0001; albumin was below normal in only 1 PLS patient, 2.8%, versus 11, 8,4%, patients in the PUMN group and 19, 4.4% patients in the classic ALS group; *p* = 0.157). Similarly, creatinine was less frequently below normal range in PLS patients (10 patients, 27.8%, compared to 62, 45.2%, in PUMN and 209, 44.2%, in classic ALS; *p* = 0.143). Phosphorus levels were never abnormal in any patient.

The progression rate in each subgroup remained unchanged during the disease course (Table S3 and Fig S2).

Only 3 PLS patients (0.05%) presented with respiratory symptoms at diagnosis (compared to 11, 0.06%, in the PUMN group and 160, 0.22%, in the classic ALS group; *p* < 0.0001). However, although FVC values declined progressively from PLS to classic ALS patients (Table S3), 10 PLS patients (22.7%) had FVC values below 70% at diagnosis, compared to 27 (17.4%) in the PUMN group and 130 (21.3%) in the classic ALS group (*p* = 0.526).

At the time of the first visit, 15 patients (26.3%) met the criteria for probable PLS, while 6 (10.6%) had already received a diagnosis of definite PLS. The remaining 36 patients (63.1%) did not meet the criteria for probable PLS at the time of their first visit, as their symptoms had started less than 2 years earlier (median diagnostic delay: 18.7 months; IQR 10.6–29.9; range: 2.5–174 months). These patients, also referred to as “early PLS,” did not differ in any of the evaluated characteristics compared to the rest of the PLS cohort (data not shown).

Patients were subsequently followed with regular visits at 1 of the ALS centers, over a median follow‐up period of 4.35 years (IQR 2.42–6.09), with a median of 12 visits (IQR 1–20). By the final follow‐up, all patients had a disease duration exceeding 2 years (thus qualifying for inclusion in this study), and the majority (43 patients, 75.4%) had surpassed 4 years. Among the remaining 14 patients who did not reach a definite PLS diagnosis, 12 were lost to follow‐up, and 2 deceased shortly after reaching the 2‐year disease duration threshold. Notably, 1 of the 15 patients classified as probable PLS at diagnosis developed LMN signs after approximately 3 years of disease progression, evolving toward a classic ALS phenotype.

**FIGURE 2:**
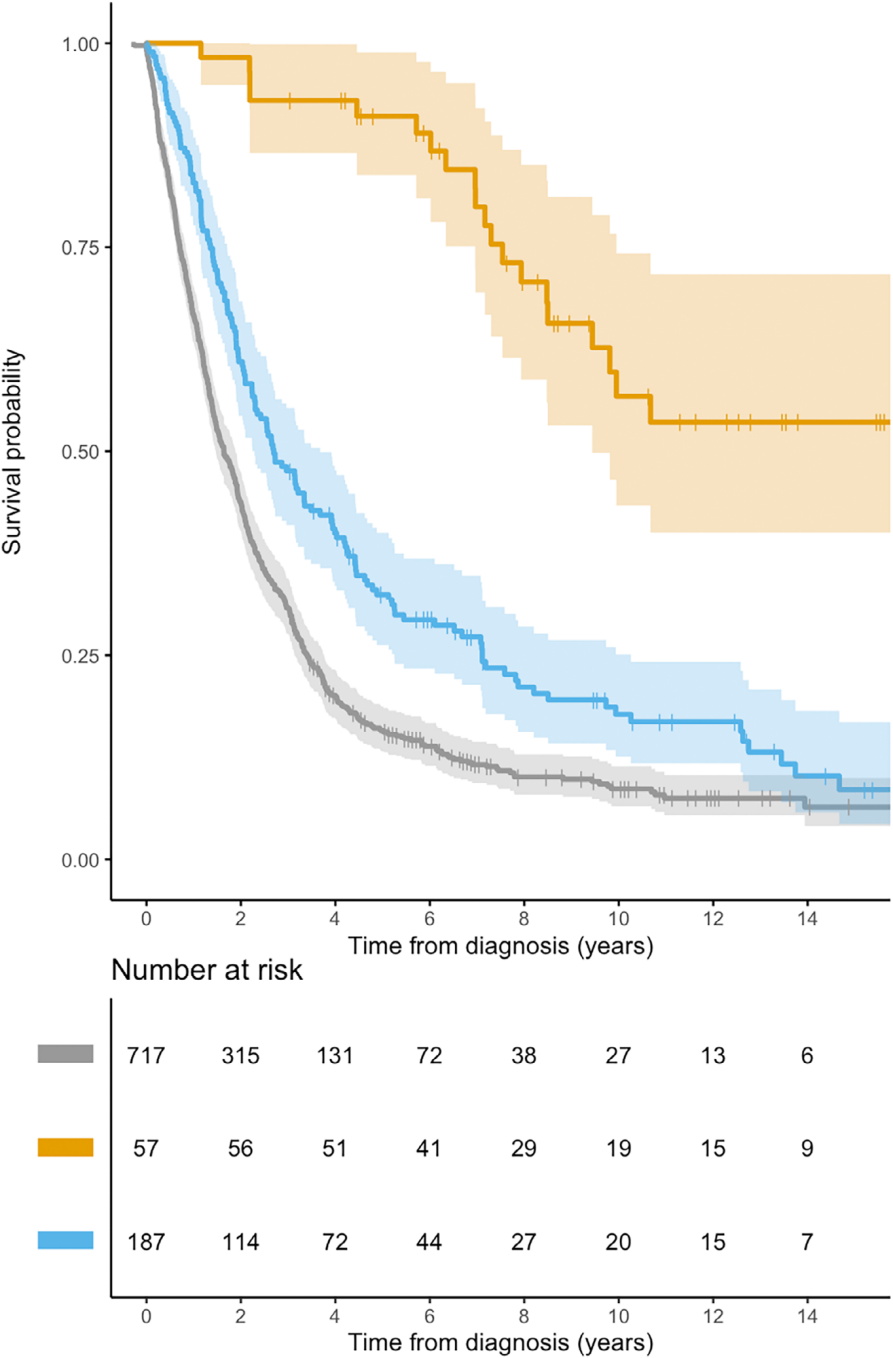
Kaplan–Meier curves and risk tables stratifies according to motor neuron phenotypes (gray = classic ALS; blue = predominant UMN; orange = primary lateral sclerosis). ALS = amyotrophic lateral sclerosis; UMN = progressive upper motor neuron. [Color figure can be viewed at www.annalsofneurology.org]

At the censoring date, the majority of PLS patients (38, 66.7%) were still alive, with a median survival of 8.3 years (IQR 5.7–12.3), significantly longer with respect to PUMN (2.7, IQR 1.4–5.8) and classic ALS patients (1.6, IQR 0.7–3.4) (*p* < 0.0001, Fig 2). This corresponded to a point prevalence of 0.89 per 100,000 inhabitants as of December 31, 2024. None of the PLS patients underwent tracheostomy; however, a significant proportion (16 patients, 38.1%) initiated non‐invasive mechanical ventilation during the disease course, and a smaller subset (6 patients, 15.8%) underwent gastrostomy placement. Age at onset (hazard ratio [HR] 1.17, 95% confidence interval [CI]: 1.05–1.33; *p* = 0.001), sex (HR for males 4.41, 95% CI: 1.24–15.6; *p* = 0.02), and FVC values at diagnosis (HR 0.95, 95%CI 0.93–0.98, *p* = 0.006) were significantly associated with PLS survival (Table S2). Accordingly, median survival was 9.9 years (IQR 6.0–10.2) among those below the median onset age and 7.2 years (IQR 4.5–7.5) among those above the median (*p* = 0.0025).

## Discussion

Using a large population‐based approach, this study described the epidemiology of PLS in a broad region of Northern Italy. Although several large studies have analyzed PLS cohorts,^12^, ^13^, ^14^, ^15^, ^16^, ^17^, ^18^, ^19^, ^20^, ^21^, ^22^, ^23^, ^24^ with 1 multicenter effort including as many as 250 patients,^25^ to our knowledge, population‐based studies on PLS were lacking.

Our findings align with previous reports on the rarity of PLS, confirming that it represents approximately 3% of all ALS cases,^19^, ^22^ with an incidence rate of 0.084/100,000 person‐years.

While some demographic and clinical characteristics were consistent with previous studies, the population‐based design of our study may have resulted in slightly different findings for other aspects. In particular, although PLS onset in our cohort never occurred before the fourth decade (consistent with previous reports^26^), we found the median age at onset to be higher (63 years; the mean age was 61.7 years) compared to 50–55 years most commonly reported in earlier studies.^12^, ^15^, ^22^, ^25^, ^26^ While a multi‐center study also found a similar higher age of onset,^16^ this discrepancy may reflect the higher probability of including younger patients in center‐based, non‐epidemiological, studies.

We also found that PLS patients were more frequently female, in contrast with most previous studies reporting a variable predominance of males,^26^ and only rarely a male‐to‐female ratio equal to 1.^15^, ^25^ Interestingly, the female predominance gradually shifted as the phenotype included increasing lower motor neuron involvement. It is well known that male predominance exists among patients with a flail arm phenotype,^27^ but, to our knowledge, evidence of such a gradient has not previously been reported. Males and females differ in many aspects, and these differences may be linked to genetics factors (unrelated to hormones), environmental factors, or hormonal influences. The fact that gain‐of‐function mutations in the *AR* gene are believed to underlie lower motor neuron involvement in Kennedy's disease^28^ could support hormonal mechanisms as a plausible explanation. However, this remains speculative at the time of this study, and *ad hoc* studies are needed.

On the other hand, we confirmed that bulbar onset is a less common presentation in PLS,^15^, ^22^ occurring in ~20% of patients. The majority of patients with bulbar onset eventually developed spinal symptoms (81.8%), and conversely, most patients with spinal onset later developed bulbar symptoms (86.9%). The study also confirmed^22^ that mutations in the main ALS‐related genes are rare, and no patients had a *C9orf72* expansion, which aligns with prior findings that such cases are very uncommon.^13^, ^29^

We did not observe any significant differences in socioeconomic status or in the frequency of the comorbidities considered between PLS patients and other motor neuron disease phenotypes.^4^ In this sense, this study did not provide any clue on the etiology of PLS.

We also confirmed that a greater degree of upper motor neuron involvement is associated with a more indolent disease course. These findings confirm that when using ALSFRSr to monitor progression in PLS, a randomized clinical trial would require either a very large sample size or a very long follow‐up period to detect a meaningful effect. This emphasizes the need for more sensitive scales^16^ or biomarkers to accurately capture ongoing damage in these patients. In this regard, we found that some muscle biomarkers (CPK and phosphorus) were significantly lower in PLS patients, also with respect to PUMN, suggesting their potential utility in differential diagnosis. Notably, 3 PLS patients showed higher levels of CPK values which suggest that the alteration of these parameters should not exclude a PLS diagnosis.^19^ However, we lack longitudinal data and we can only hypothesize that the slow progression of the disease limits the usefulness of these markers in reflecting the upper motor neuron damage over time.

Also, PLS patients did not show a decrease in BMI from symptom onset to diagnosis.^25^

The population‐based design also allowed us to include a substantial number of early PLS patients. Early PLS has been rarely described and poses a diagnostic challenge, as lower motor neuron signs may still emerge within the first 4 years of disease progression.^2^, ^30^ However, as also confirmed by our findings, these patients do not exhibit any distinct demographic or clinical characteristics at the time of diagnosis compared to other PLS patients.

One patient initially diagnosed with probable PLS developed lower motor neuron signs 3 years after symptom onset, leading to a revised diagnosis of ALS. Conversion from PLS to ALS is a known occurrence and has been reported with varying frequencies. In some studies, conversion was observed only within the first 4 years,^15^, ^24^, ^25^ as in our case, while in others it occurred later in the disease course, even after decades,^15^, ^17^, ^23^, ^24^, ^31^ raising questions about the true conversion rate if longer survival and extended follow‐up were available.^31^ This finding—together with the gradient observed across phenotypes in age at onset and male‐to‐female ratio—raises the question of whether PLS should be considered a distinct disease from ALS or rather the extreme end of a single spectrum encompassing both.^2^, ^15^ In favor of a unified disease spectrum, some studies (though not all^2^, ^32^, ^33^) showed the presence of neuropathological features in the motor cortex, such as TDP‐43 cytoplasmic inclusions and Bunina bodies,^32^, ^33^, ^34^, ^35^ which resemble those found in ALS. Additionally, lower motor neurons of PLS patients seem to display subclinical neuropathological and neurophysiological abnormalities.^23^, ^36^ This may ultimately reflect an ontological issue: how much subclinical lower motor neuron involvement can be tolerated before a case should be classified as PUMN rather than PLS? It is likely that only the development of a reliable biomarker capable of distinguishing PLS from ALS (and HSP) will resolve this debate.^4^

Finally, we confirmed that median survival among PLS patients is significantly longer than in classic ALS, exceeding 8 years.^15^, ^22^, ^25^ We also found that age at onset is a predictor of survival in PLS, whereas the site of onset was not associated with prognosis.^22^ Consistent with most ALS phenotypes,^37^ male patients showed a worse prognosis. Interestingly, and in contrast to a previous study,^22^ some patients presented with respiratory symptoms at diagnosis, and a greater number showed abnormal FVC values, which were significantly associated with shorter survival. We also confirmed that a proportion of PLS patients require non‐invasive mechanical ventilation during the disease course,^22^ and that a small percentage underwent gastrostomy placement.^22^, ^25^ However, no patients in our cohort underwent tracheostomy.^25^

The population‐based design, the deep phenotyping and the long‐term follow‐up of the patients included represent the major strengths of this study. However, some limitations should be acknowledged, the most important being its retrospective nature. This limited our ability to systematically and longitudinally collect certain data, such as the presence of extra‐motor signs in PLS.^2^, ^14^, ^23^, ^26^, ^38^, ^39^ Blood neurofilaments measurements were not available, since the study period preceded their implementation as a biomarker for ALS, preventing us from evaluating their diagnostic and prognostic utility. A comprehensive genetic screening for all known HSP‐related genes was not conducted in all patients. Lastly, while a higher age of onset and a predominance of female patients appear to be among the key findings of this study, it should be noted that the small sample size may limit the generalizability of these results.

In conclusion, we confirmed that PLS is a rare disease. Patients are generally younger, although the age at onset in our cohort was higher than previously reported. We also observed a female predominance, which may reflect a broader sex‐related gradient in lower motor neuron involvement, despite further studies are needed. PLS is characterized by a slow progression throughout the entire disease course, limiting the feasibility of randomized clinical trials when using ALSFRSr score changes as the primary outcome. Finally, in addition to age at onset, both male sex and respiratory function at diagnosis were associated with survival, a finding that may be valuable for prognostic counseling in clinical practice.

## Author Contributions

R.V., E.M., A.Cal., and A.Chi. contributed to the conception and design of the study. G.P., A.Can., U.M., M.G., F.P., S.C., A.M., F.D'O., G.M., S.G., E.D'A., L.M., F.D.M., and C.M. contributed to acquisition and analysis of data. R.V. contributed to drafting the text and preparing the figures.

## Potential Conflicts of Interest

Nothing to report.

## Supporting information


**Table S1.** Annual crude incidence rate of primary lateral sclerosis during the study period.
**Table S2**. Results of the Cox regression analysis evaluating predictors of survival in primary lateral sclerosis.
**Tables S3**. Demographical and clinical characteristics of patients across the motor neuron phenotypes
**Figure S1**. Crude incidence rate of Primary Lateral Sclerosis across three consecutive 5‐year intervals.
**Figure** S**2**. ΔFRS at the end of the follow‐up across different motor neuron disease phenotypes.

## Data Availability

Data are available upon reasonable request by interested researchers.
