# Supplementary material for: The Epidemiology of Primary Lateral Sclerosis: Results from a Population‐Based Cohort
Source: Ann Neurol. 2025 Nov 28;99(3):606–13. doi: 10.1002/ana.78105 (PMC12954144; doi:10.1002/ana.78105)
Supplement: Supplementary file 1 — Table S1. Annual crude incidence rate of primary lateral sclerosis during the study period. Table S2. Results of the Cox regression analysis evaluating predictors of survival in primary lateral sclerosis. Tables S3. Demographical and clinical characteristics of patients across the motor neuron phenotypes Figure S1. Crude incidence rate of Primary Lateral Sclerosis across three consecutive 5‐year intervals. Figure S2. ΔFRS at the end of the follow‐up across different motor neuron disease phenotypes. [file ANA-99-606-s001.doc]

**Supplementary material.**

The panel of HSP-related genes was tested in 5 (8.7%) patients and included the following genes: *ABCD1, ACBD5, ACO2, ADAR, AFG3L2, ALDH18A1, ALS2, AMPD2, AP4B1, AP4E1, AP4M1, AP4S1, AP5Z1, ARL6IP1, ARSI, ATAD3A, ATL1, ATL3, ATP13A2, ATP2B4, B4GALNT1, BICD2, BSCL2, C12ORF65, C19ORF12, C9ORF72, CAPN1, CAV1, CCT5, COASY, CPT1C, CYP27A1, CYP2U1, CYP7B1, DARS2, DDHD1, DDHD2, DSTYK, DYNC1H1, ENTPD1, EPT1, ERLIN1, ERLIN2, EXOSC3, EXOSC8, FA2H, FARS2, FBXO7, FLRT1, GAD1, GBA2, GCH1, GDAP1, GDAP2, GJA1, GJC2, GLB1, GPT2, HSPD1, IBA57, IFIH1, IFRD1, KCNA2, KIAA0196, KIDINS220, KIF1A, KIF1C, KIF5A, KLC2, KLC4, KY, LICAM, LYST, MAG, MARS, MARS2, MFN2, MTHFR, MTPAP, NIPA1, NPC1, NT5C2, OPA1, OPA3, PANK2, PEX16, PGAP1, PLA2G6, PLP1, PNPLA6, POLR3A, RAB3GAP2, REEP1, REEP2, RNASEH2B, RTN2, SACS, SAMHD1, SERAC1, SETX, SLC16A2, SLC33A1, SOX10, SPAST, SPG11, SPG20, SPG21, SPG7, SYNE1, TECPR2, TFG, TRMT5, TRPV4, TTC19, TUBB2A, TUBB4A, UBQLN2, UCHL1, USP8, VAMP1, VCP, VPS13D, VPS37A, VRK1, WDR48, WWOX, ZFR, ZFYVE26, ZFYVE27*.

**Supplementary table 1.** Annual crude incidence rate of Primary Lateral Sclerosis during the study period.

| **Year** | **Patients (n)** | **Population at risk** | **Crude incidence rate** |
| --- | --- | --- | --- |
| 2007 | 2 | 4430654 | 0.04 |
| 2008 | 5 | 4483931 | 0.11 |
| 2009 | 7 | 4515625 | 0.15 |
| 2010 | 2 | 4528529 | 0.04 |
| 2011 | 3 | 4538040 | 0.07 |
| 2012 | 6 | 4544050 | 0.13 |
| 2013 | 5 | 4553767 | 0.11 |
| 2014 | 1 | 4549169 | 0.02 |
| 2015 | 1 | 4531534 | 0.02 |
| 2016 | 6 | 4511256 | 0.13 |
| 2017 | 4 | 4497025 | 0.09 |
| 2018 | 2 | 4476124 | 0.04 |
| 2019 | 5 | 4454218 | 0.11 |
| 2020 | 6 | 4436251 | 0.14 |
| 2021 | 2 | 4399034 | 0.05 |

|  |  |  |  |
| --- | --- | --- | --- |
|  |  |  |  |
|  |  |  |  |
|  |  |  |  |

**Supplementary table 2.** Results of the Cox regression analysis evaluating predictors of survival in Primary Lateral Sclerosis.

|  | **HR (95% CI)** | **p.value** |
| --- | --- | --- |
| Sex, M | 7.72 (1.64-36.41) | 0.01 |
| Onset site, spinal | 0.52 (0.09-3.03) | 0.46 |
| Onset age | 1.13 (1.02-1.25) | 0.01 |
| Diagnostic delay | 1.03 (1.01-1.06) | 0.01 |
| ΔFRS at diagnosis | 7.02 (0.66-74.32) | 0.11 |
| ΔBMI at diagnosis | 1.52 (0.55-4.22) | 0.42 |
| FVC | 0.95 (0.91-0.98) | <0.001 |

Diagnostic delay = number of months from symptom onset to diagnosis; ΔFRS at diagnosis = (48 – ALSFRS-r score at diagnosis)/diagnostic delay; ΔBMI at diagnosis = premorbid BMI - BMI at diagnosis/diagnostic delay.

**Supplementary table S3.** Demographical and Clinical Characteristics of Patients across the Motor Neuron Phenotypes

| Parameter | Classic ALS (n = 720) | PUMN (n = 187) | PLS (n = 57) | *p*-Value |
| --- | --- | --- | --- | --- |
| Sex, M (%) | 448 (62.2) | 111 (59.4) | 21 (36.8) | 0.001 |
| Onset age, median (IQR) | 66.9 (58.8–73.9) | 64.3 (54.4–71.7) | 63.5 (54.9–70.4) | <0.001 |
| Onset site, n (%) |  |  |  |  |
| Bulbar | 0 (0.0) | 0 (0.0) | 11 (19.3) | <0.001 |
| Lower limbs | 317 (44.0) | 156 (83.4) | 42 (73.7) |  |
| Upper limbs | 403 (56.0) | 31 (16.6) | 4 (7.0) |  |
| Onset side, n (%) |  |  |  |  |
| Bilateral | 181 (25.3) | 78 (41.9) | 28 (60.9) | <0.001 |
| Unilateral | 535 (74.7) | 108 (59.1) | 18 (39.1) |  |
| Diagnostic delay, median (IQR) | 9.6 (6.5–13.7) | 11.6 (7.5–21.8) | 18.7 (10.6–29.9) | <0.001 |
| Marital status, n (%) |  |  |  |  |
| Unmarried | 68 (9.6) | 15 (8.2) | 5 (8.9) | 0.991 |
| Married | 525 (74.4) | 141 (77.5) | 42 (75.0) |  |
| Divorced | 26 (3.7) | 7 (3.8) | 2 (3.6) |  |
| Separated | 16 (2.3) | 2 (1.1) | 1 (1.8) |  |
| Widow/er | 71 (10.1) | 17 (9.3) | 6 (10.7) |  |
| Educational level, n (%) |  |  |  |  |
| No formal education | 20 (2.9) | 7 (3.9) | 1 (1.8) | 0.861 |
| Elementary school | 211 (30.2) | 50 (27.9) | 13 (23.2) |  |
| Middle school | 220 (31.5) | 62 (34.6) | 17 (30.4) |  |
| High school | 140 (20.1) | 34 (19.0) | 13 (23.2) |  |
| Graduate | 60 (8.6) | 13 (7.3) | 5 (8.9) |  |
| Technical school | 47 (6.7) | 13 (7.3) | 7 (12.5) |  |
| Smoking status, n (%) |  |  |  |  |
| Ex-smoker | 233 (33.5) | 64 (36.2) | 17 (31.5) | 0.354 |
| Never smoker | 310 (44.6) | 84 (47.5) | 29 (53.7) |  |
| Current smoker | 152 (21.9) | 29 (16.4) | 8 (14.8) |  |
| Arterial hypertension, n (%) | 350 (48.9) | 89 (48.1) | 25 (47.2) | 0.959 |
| Diabetes type II, n (%) | 90 (12.5) | 17 (9.2) | 4 (7.0) | 0.247 |
| Thyroid diseases, n (%) | 76 (10.6) | 20 (10.7) | 9 (15.8) | 0.472 |
| Tumor, n (%) | 90 (12.5) | 27 (14.4) | 10 (17.5) | 0.473 |
| Familial history, positive (%) | 68 (9.6) | 12 (6.7) | 0 (0) | 0.031 |
| *C9orf72*, expanded (%) | 47 (7.3) | 6 (3.6) | 0 (0.0) | 0.038 |
| Missing | 78 (10.8) | 21 (11.2) | 8 (14.0) |  |
| *SOD1*, mutated (%) | 13 (2.0) | 5 (3.0) | 0 (0.0) | 0.423 |
| Missing | 83 (11.5) | 22 (11.7) | 9 (14.0) |  |
| *TARDBP*, mutated (%) | 7 (1.1) | 3 (1.8) | 0 (0.0) | 0.552 |
| Missing | 83 (11.5) | 22 (11.7) | 9 (14.0) |  |
| *FUS*, mutated (%) | 3 (0.5) | 0 (0.0) | 0 (0.0) | 0.605 |
| Missing | 83 (11.5) | 22 (11.7) | 9 (14.0) |  |
| CPK, median (IQR) | 170.0 (97.0–273.5) | 128.0 (83.8–172.5) | 82.0 (62.5–133.5) | <0.001 |
| Missing | 281 (39.0) | 55 (29.4) | 22 (38.6) |  |
| Creatinine, median (IQR) | 0.74 (0.60–0.90) | 0.74 (0.61–0.91) | 0.79 (0.68–0.92) | 0.381 |
| Missing | 247 (34.3) | 50 (26.7) | 21 (36.8) |  |
| Phosphorus, median (IQR) | 1.10 (1.00–1.20) | 1.10 (1.00–1.20) | 1.00 (0.90–1.10) | 0.013 |
| Missing | 397 (55.1) | 94 (50.2) | 27 (47.4) |  |
| Albumin, median (IQR) | 4.30 (4.00–4.60) | 4.30 (4.00–4.60) | 4.41 (4.30–4.62) | 0.159 |
| Missing | 289 (40.1) | 56 (29.9) | 21 (36.8) |  |
| ΔBMI at diagnosis, median (IQR) | 0.15 (0–0.67) | 0.08 (0–0.43) | 0 (0–0.09) | <0.001 |
| ALSFRSr total score at diagnosis, median (IQR) | 41 (36–45) | 41 (36–44) | 43 (40–45) | 0.024 |
| Missing | 42 (5.8) | 15 (8.0) | 5 (8.8) |  |
| ΔFRS at diagnosis, median (IQR) | 0.71 (0.38–1.39) | 0.61 (0.30–1.13) | 0.29 (0.19–0.51) | <0.001 |
| Missing | 42 (5.8) | 15 (8.0) | 5 (8.8) |  |
| FVC, median (IQR) | 90.0 (73.0–100.0) | 92.0 (79.5–100.0) | 98.0 (73.0–100.0) | 0.352 |
| Missing | 111 (15.4) | 32 (17.1) | 13 (22.8) |  |
| NIMV, yes (%) | 324 (48.1) | 70 (40.2) | 16 (38.1) | 0.099 |
| Gastrostomy, yes (%) | 157 (24.0) | 48 (28.6) | 6 (15.8) | 0.206 |
| Tracheostomy, yes (%) | 82 (12.6) | 18 (10.8) | 0 (0.0) | 0.059 |

*Note:* For detailed definitions of the classic and PUMN phenotypes, refer to Reference [9].

ALSFRS-r = revised ALS Functional Rating Scale; ΔBMI at diagnosis = premorbid BMI-BMI at diagnosis/diagnostic delay; BMI = body mass index; diagnostic delay = number of months from symptom onset to diagnosis; ΔFRS at diagnosis = (48 – ALSFRS-r score at diagnosis)/diagnostic delay; FRS = ALS Functional Rating Scale; IQR = interquartile range; NIMV = non-invasive mechanical ventilation; PLS = primary lateral sclerosis; PUMN = predominant UMN phenotype.

**Supplementary figure 1.** Crude incidence rate of Primary Lateral Sclerosis across three consecutive 5-year intervals**.**

**
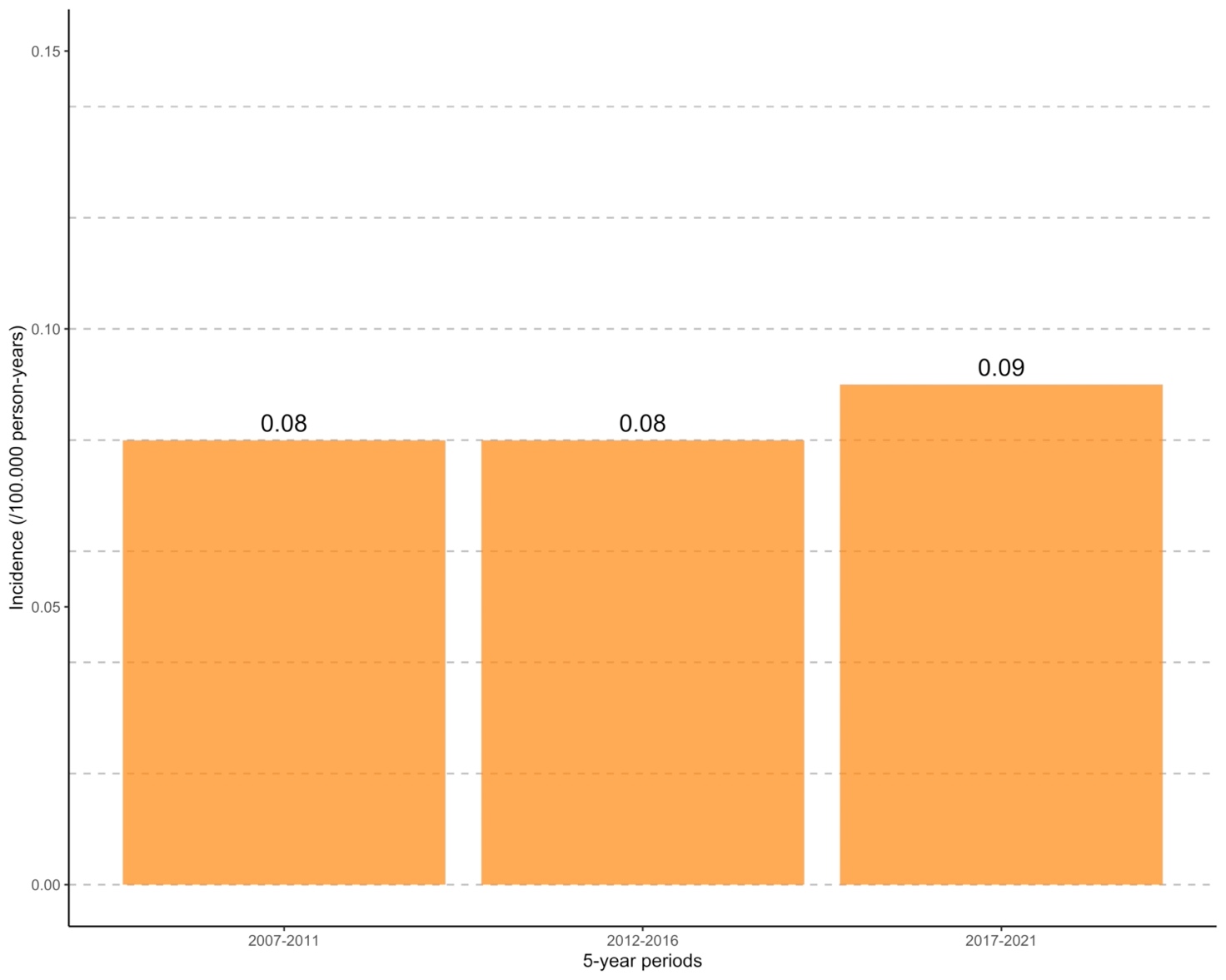
**

**Supplementary figure 2.** ΔFRS at the end of the follow-up across different motor neuron disease phenotypes.


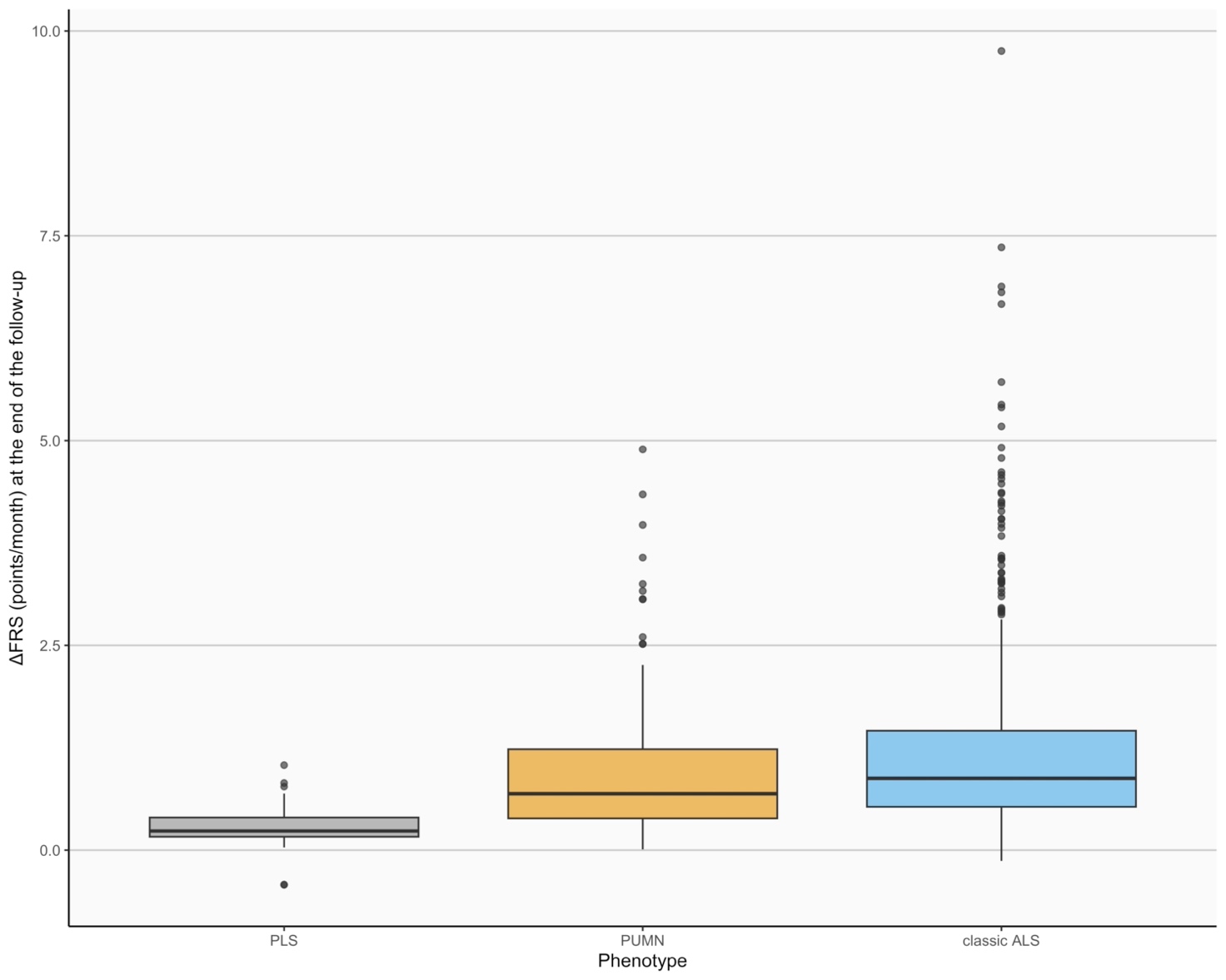


PLS: Primary Lateral Sclerosis. PUMN: predominant UMN phenotype.
